# Inaccurate Saccades and Enhanced Vestibulo-Ocular Reflex Suppression during Combined Eye–Head Movements in Patients with Chronic Neck Pain: Possible Implications for Cervical Vertigo

**DOI:** 10.3389/fneur.2017.00023

**Published:** 2017-01-30

**Authors:** Janine L. Johnston, Pierre M. Daye, Glen T. D. Thomson

**Affiliations:** ^1^CIADS Research, University of Manitoba, Winnipeg, MB, Canada; ^2^Department of Ophthalmology, University of Manitoba, Winnipeg, MB, Canada; ^3^Department of Medicine, University of Manitoba, Winnipeg, MB, Canada; ^4^Vision and Natural Computation Group, Vision Institute, Paris, France; ^5^Department of Family Medicine, University of Manitoba, Winnipeg, MB, Canada

**Keywords:** VOR suppression, cervical vertigo, combined eye–head saccades, gaze kinematics, neck pain

## Abstract

**Background:**

The primate ocular motor system is designed to acquire peripheral targets of interest by coordinating visual, vestibular, and neck muscle activation signals. The vestibulo-ocular reflex (VOR) is greatly reduced at the onset of large eye–head (gaze) saccades and resumes before the end of the saccades to stabilize eye-in-orbit and ensure accurate target acquisition. Previous studies have relied on manipulating head movements in normal individuals to study VOR suppression and gaze kinematics. We sought to determine if reduced head-on-trunk movement alters VOR suppression and gaze accuracy similar to experiments involving normal subjects and if intentionally increasing head and neck movement affects these dynamics.

**Methods:**

We measured head and gaze movements using magnetic search coil oculography in eight patients with cervical soft tissue disorders and seven healthy subjects. All participants made horizontal head-free saccades to acquire a laser dot target that stepped pseudorandomly 30–65° to either side of orbital mid-position, first using typical head and eye movements and again after being instructed to increase head amplitudes as much as possible.

**Results:**

Compared to healthy subjects, patients made smaller head movements that contributed only 6% to total gaze saccade amplitudes. Head movements were also slowed, prolonged, and delayed. VOR suppression was increased and prolonged. Gaze saccades were inaccurate and delayed with long durations and decreased peak velocities.

**Conclusion:**

In patients with chronic neck pain, the internal commands issued for combined eye–head movements have large enough amplitudes to create accurate gaze saccades; however, because of increased neck stiffness and viscosity, the head movements produced are smaller, slower, longer, and more delayed than they should be. VOR suppression is disproportionate to the size of the actual gaze saccades because sensory feedback signals from neck proprioceptors are non-veridical, likely due to prolonged coactivation of cervical muscles. The outcome of these changes in eye–head kinematics is head-on-trunk stability at the expense of gaze accuracy. In the absence of vestibular loss, the practical consequences may be dizziness (cervical vertigo) in the short term and imbalance and falls in the long term.

## Introduction

When navigating through our world, we need to quickly identify targets of interest or obstacles that may obstruct our passage. The primate ocular motor system is designed to stabilize vision to accomplish these goals. During most natural head movements, clear vision results from tightly coordinated signals originating in visual, vestibular, and neck muscle activation systems. Within the central 50° of fixation, saccades can be made using the eyes alone, but to acquire a more peripheral target, eye movements must be combined with head movements. The vestibulo-ocular reflex (VOR) reflexively produces compensatory eye movements in the direction opposite to head movements, thereby stabilizing vision. However, during combined eye–head (gaze) saccades, the gaze and head can move in the same direction and the VOR must be suppressed. This typically occurs at the onset of the gaze saccade (1). Likewise, the head has greater inertia and needs time to contribute to the gaze saccade, so VOR suppression correlates with the duration of gaze displacement (2). These kinematics are accomplished by a gaze feedback controller that varies head and gaze velocities and durations in order to minimize signal noise and reduce gaze error (3, 4). The cerebellum is involved in this controller, ensuring gaze and head movement accuracy, especially for large gaze shifts when the VOR is suppressed (2). Finally, VOR cancellation signals occur when there is activation of neck proprioception but only when that activation matches what is expected from the command to the neck muscles (5).

Prior studies have examined gaze accuracy during combined eye–head movements while modifying head movements by increasing head inertia (3, 6) or braking the head [e.g., Ref. (7)]. We sought to utilize individuals who had restricted head-on-trunk movements on the basis of cervical muscle pain. These individuals often complain of dizziness which has no known physiologic basis, although sensorimotor mismatch has recently been speculated as a cause since intended head movements and associated vestibular signals are smaller than expected (8). These subjects also have increased imbalance and predisposition to falls in the absence of vestibular loss (9). By comparing them to healthy subjects without restricted neck movements, we sought to determine whether reduced head-on-trunk movements affect VOR suppression and gaze accuracy during combined eye–head movements and whether intentionally increasing head and neck movement amplitudes could affect these dynamics.

## Participants and Methods

We recorded head and gaze movements in seven healthy subjects (controls) without neck pain (mean age 32 years; range 24–40 years). These were compared to eight individuals (patients) (mean age 38 years; range 22–58 years) who had restricted neck movements on the basis of chronic cervical muscle pain characterized by aching and stiffness of neck and shoulder girdle muscles (mean duration of pain 8 years; range 1–25 years). Exclusion criteria for both patients and healthy subjects were the same and included a past or current history of vertigo, vestibular, or cerebellar disorders, head trauma, whiplash, or cervical dystonia. All subjects underwent neuro-otological examination, and all patients had a full musculoskeletal assessment. No additional tests were performed on the subjects or the patients to assess the VOR behavior prior to this study. There was no bony pathology, such as ankylosing spondylitis, cervical disk disease, or significant osteoarthritis, to restrict passive cervical range of motion, which was normal for all patients. No patient or healthy subject was using major tranquilizers or sedatives, had past or current drug or alcohol abuse, external eye disease, or abnormal general neurologic examination, and no patient had been treated with botulinum toxin. Measurements of the VOR using sinusoidal *en bloc* stimulation with an imaginary stationary target in darkness at 0.025 and 0.05 Hz were assured to be within normal limits for patients as were saccade metrics for horizontal head-fixed saccades. Low oscillating frequencies were used to increase the sensitivity of sinusoidal rotational testing and enhance the likelihood of detecting abnormalities across a broader range of vestibular function (10, 11).

Patients and control subjects had horizontal head and gaze positions recorded using a magnetic search technique (CNC Engineering, Seattle, WA, USA), after informed consent was obtained. The study was approved by the University of Manitoba Ethics committee and conformed to the Declaration of Helsinki. A scleral contact ring containing a coil was placed on one eye measuring gaze movements, and the second coil was taped firmly to the forehead measuring head movements. Each participant sat in the vestibular chair in dim illumination and made horizontal head-free saccades to a laser dot target that stepped 30–65° pseudorandomly to right or left of mid-position at intervals of 0.25–3.0 s. The target was a 0.5-mW He–Ne laser dot subtending in angle of 0.29° and projected onto a featureless screen at a distance of 1.63 m from the nasion. Initially, subjects were not instructed as to how to move their heads; these represented typical head movements. The same test was repeated with instructions to move their heads as much as possible in order to acquire the targets. Each trial consisted of 20 target jumps.

Source coils were earth fixed and measured 182.88 cm in diameter; each subject was centered within the linear range of the magnetic field. Translational head movements were not measured during head-free saccades. While it is possible that some subjects used horizontal head translations to assist in acquiring the visual target during large gaze shifts, the amplitude of these translations is usually small (12) and would not have affected the accuracy of the rotational head or gaze signals on which results were based. We verified the insensitivity of our rotational measurements to translation by measuring the difference in rotational accuracy over a two-dimensional grid within the center 36 cm^3^ of the magnetic search coil apparatus. Because the large amplitude horizontal target steps may have occasionally occurred outside the limits of linearity for the magnetic search coil apparatus, we also verified linearity between +70° and −70° horizontal rotation.

Horizontal target, gaze (eye-in-space), and head position signals were filtered (passband 1–40 Hz, sixth-order Bessel filter) and then digitized on line at 200 Hz. Target, gaze, eye (eye-in-orbit), and head position were digitally smoothed using a 9-point smoothing function with an effective low passed cutoff of 45 Hz. Horizontal gaze, eye, and head position signals were differentiated using a 2-point central differentiation algorithm (13) to yield gaze, eye, and head velocity.

Saccades were identified when gaze velocity exceeded 40°/s for longer than 0.03 s. The saccade terminated when its velocity dropped below 5°/s. Second gaze saccades, if they occurred, were identified in the same way as first saccades. Due to the greater inertia of the head, the onset of head saccades was identified when head velocity exceeded 5°/s for longer than 0.03 s. When the head velocity fell below 5°/s, the head saccade terminated.

Gaze (head) saccade latency is the difference between onset of target movement and onset of gaze (head) movement. The accuracy of gaze saccades is described as the gain, which is the ratio of the amplitude of the first (total) gaze saccade to the target amplitude. The VOR gain is the ratio of eye velocity to head velocity. We evaluated the VOR gain at the offset of the first saccade because the suppression mechanism is normally not active at that instant (14). The head contribution to the gaze saccade is the amplitude of the head movement between its onset and the termination of the gaze saccade.

### Statistical Analysis

Intra-subject data were analyzed using Wilcoxon matched-pair signed rank test. Group data between normal subjects and patients were analyzed using non-paired Mann–Whitney test with Welch’s correction, when appropriate. Comparison of values of duration and velocity based on gaze saccade amplitudes and head amplitudes was done using linear analysis comparing slopes and elevations. Correlations were performed using Spearman correlation for non-parametric data and Pearson *r* correlation for parametric data. For all statistical analyses, a two-sided value of *p* ≤ 0.05 was considered significant. Statistical figures were created with GraphPad Prism 5.0 (La Jolla, CA, USA).

## Results

### Control Subjects

The kinematics of combined eye–head movements in healthy subjects were similar to those described previously (3, 15, 16). When asked to do so, control subjects made significantly larger head movements in order to acquire peripheral targets (*p* < 0.0001) (Table 1). Likewise, with increasing head movements, the head contribution to the gaze displacement increased significantly (*p* = 0.0002) (Table 1). When subjects were asked to make larger head movements, head movement durations increased (*F* = 6.577; *p* = 0.0136) and peak head velocities were significantly faster (*F* = 9.681; *p* = 0.0026).

**Table 1:** **Head, gaze, and vestibulo-ocular reflex (VOR) comparisons**.

	Controls		Patients	
	Typical HM	Larger HM	Typical HM	Larger HM
Ratio of head amplitude to gaze amplitude	0.29 (0.03)	0.60 (0.04)	0.12 (0.01)***	0.38 (0.02)***
Ratio of head contribution to gaze amplitude	0.20 (0.02)	0.36 (0.04)	0.06 (0.01)***	0.19 (0.01)***
VOR gain	−0.62 (0.07)	−0.83 (0.05)	0.02 (0.11)***	−0.54 (0.05)***
First gaze saccade gain	0.97 (0.01)	0.95 (0.01)	0.87 (0.01)***	0.92 (0.01)*
Total gaze saccade gain	1.03 (0.01)	1.01 (0.01)	0.93 (0.01)***	0.94 (0.01)***
Gaze saccade latency (ms)	182 (8)	159 (6)	233 (14)***	234 (11)***
Δ Gaze to head onset (ms)	51 (7)	24 (6)	84 (18)*	22 (5)

*Values are means with SE in brackets*.

*Statistical values are for comparisons between patients and control subjects*.

*p < 0.05.

***p < 0.0001.

*VOR gain is the ratio of eye velocity to head velocity at the end of the first saccade during combined eye–head tracking*.

*Δ Gaze to head onset is the difference between the onset of the gaze saccade and the onset of the subsequent head movement*.

*HM, head movements*.

**Figure 1:**
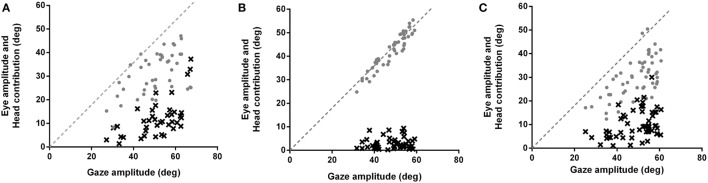
**Eye movement amplitudes (gray circles) and head contribution amplitudes (black x’s) during combined eye–head (gaze) shifts of 45–65° amplitudes in (A) healthy subjects, (B) patients making typical head movements, and (C) patients making larger head movements**. Dashed lines have slopes of 1. In healthy subjects **(A)**, the head starts contributing to the gaze saccade earlier (about 25–30°), similar to when initial eye-in-orbit position is aligned with or in the same direction as the intended gaze saccade. Patients **(B)** make gaze shifts that are almost entirely composed of eye-in-orbit movements, with limited head contribution (6%) even for saccades greater than 50°. This is similar to when the initial eye position is deviated in a direction away from the direction of the intended saccades. When patients consciously increase gaze amplitudes **(C)**, there is increasing head contribution (19%) and decreasing eye-in-orbit contribution to the gaze saccade, similar to control subjects making typical head movements (20%).

Gaze saccades remained accurate for both typical head movements and intentionally larger head movements (*p* = 0.1124) (Table 1). This is consistent with the proposition that eye movements are a consequence of the control of gaze and head trajectories through two feedback loops (2). Asymptotic peak velocity was faster for gaze saccades made with intentionally larger head movements, but this increase did not reach statistical significance (*p* = 0.4035) (Table 2). However, gaze saccade durations were shorter (*F* = 0.5623, *p* = 0.0202), as would be expected with larger head movements (15). Gaze saccades were made with shorter latencies for intentionally larger head movements (*p* < 0.0001). Gaze onset always preceded head movement onset because of greater head inertia. There was also significantly less time between gaze onset and head movement onset (*p* < 0.0001) (Table 1), which decreased as a function of gaze amplitude for typically sized (Spearman *r* = −0.3009, *p* = 0.0472) and intentionally larger head movements (Spearman *r* = −0.3874, *p* = 0.0094), similar to non-human primates (17). That is, with increasing gaze amplitudes, gaze and head movements both began faster and head movements factored into gaze saccades earlier, with increasing head and decreasing eye-in-orbit contributions to the gaze saccades (Figure 1A). Although exact eye-in-orbit positions at the beginning of the gaze movement were not recorded, this is consistent with healthy subjects having their eyes directly on target or deviated toward the direction of the gaze shift (16).

**Table 2:** **Gaze saccade asymptotic peak velocity comparisons**.

Group	Gaze peak velocity (°/s)	
	Typical HM	Larger HM
Controls	586 (59)	675 (88)
Patients	486 (41)	475 (40)*

*Values are means with SE in brackets*.

*p = 0.0435 for patients compared to controls.

*HM, head movements*.

**Figure 2:**
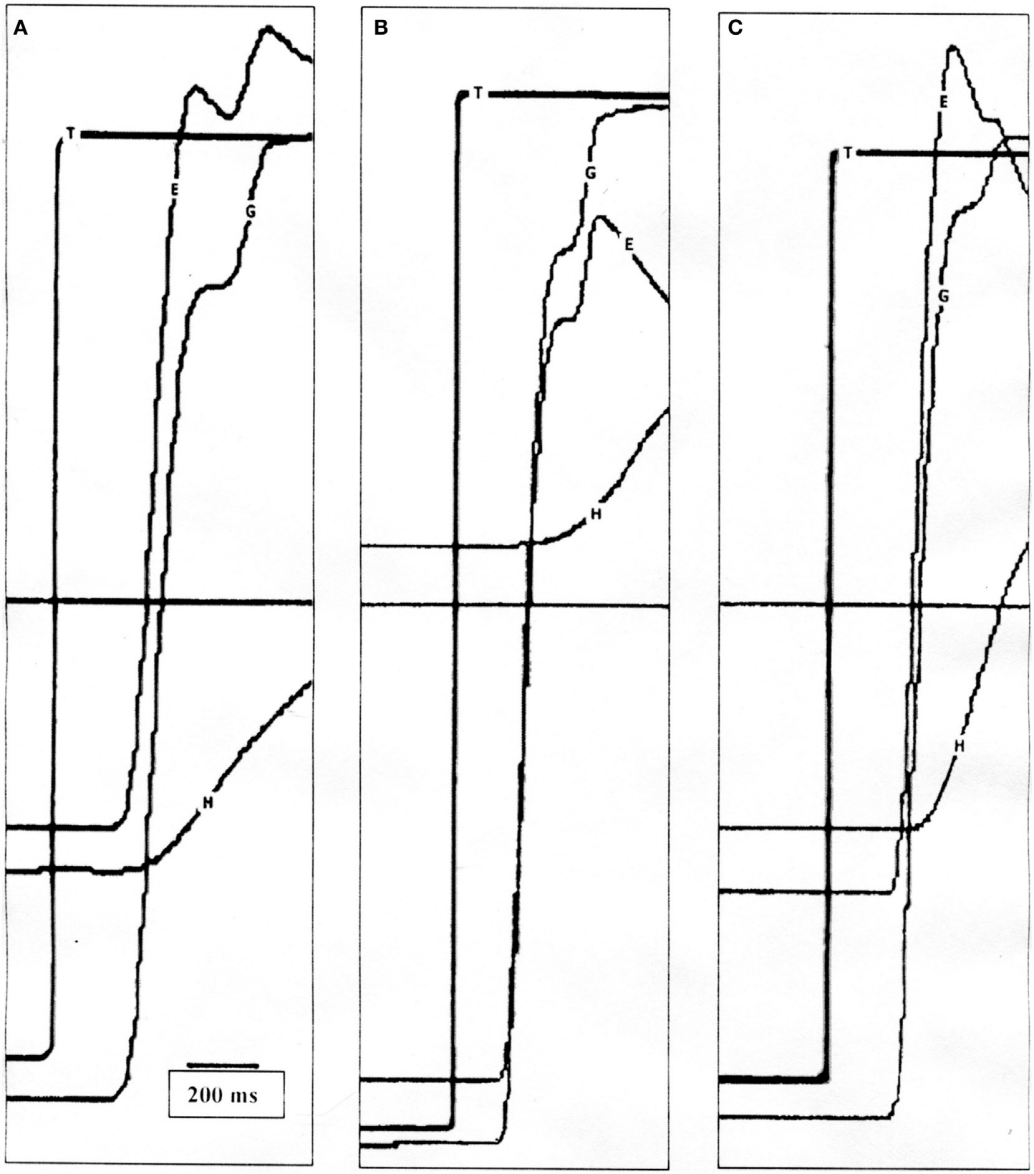
**(A)** Position tracings of a control subject making a 50° saccade with typical head movement. The vestibulo-ocular reflex (VOR) is active at the end of the gaze saccade and the eye-in-orbit counter-rotates to maintain target fixation. **(B)** Position tracings of a patient making a 60° saccade with typical head movement. The first gaze saccade is hypometric, and the VOR is suppressed allowing the gaze to move in the same direction as the head, followed by another saccade. Only when the target is acquired does the VOR become active. **(C)** Position tracings of the same patient making a 50° saccade with intentionally increased head movement amplitude. Similar to the healthy subject, the VOR is near unity at the end of the first saccade. T, target; G, gaze (eye-in-space); E, eye-in-orbit; H, head movement.

When making combined eye–head saccades, the VOR must be disabled to prevent compensatory eye-in-orbit movements opposite to the gaze shifts. VOR suppression is greatest early in the gaze shift and diminishes near the end of the movement so that the VOR is fully restored by the end of the eye–head movement (1, 14). This can be seen in ocular motor tracings (Figure 2A), which show the VOR to be active at the end of the gaze saccade with counter-rotation of the eye-in-orbit, thereby maintaining target fixation. Typically, VOR gain decreases as function of increasing gaze amplitude (1), and for healthy subjects, there was a negative correlation between VOR gain and gaze amplitude (Pearson *r* = −0.2224, *p* = 0.0352). Likewise, increased gaze accuracy correlated strongly with greater VOR suppression (Pearson *r* = −0.2965, *p* = 0.0045). Finally, the degree of VOR suppression and gaze duration should be correlated (2). This occurred for both typical (Pearson *r* = −0.3749, *p* = 0.0112) and enhanced (Pearson *r* = −0.7982, *p* < 0.0001) head movement conditions.

There was a trend to negative correlation between gaze saccade latency and VOR gain, but it did not reach significance for either typical (Pearson *r* = −0.2729, *p* = 0.0627) or larger (Pearson *r* = −0.2930, *p* = 0.0536) head movements. That is, there appeared to be less VOR suppression when gaze saccades began faster.

### Patients

**Figure 3:**
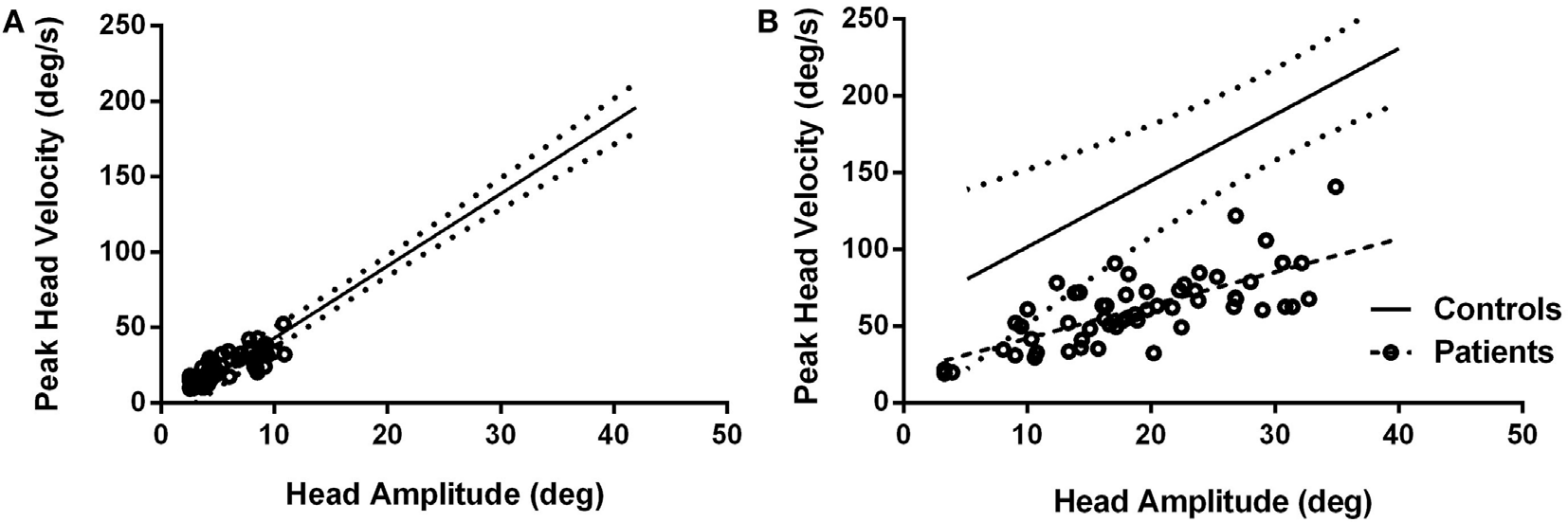
**Peak head velocities for healthy subjects and patients during typical head movements (A) and larger head movements (B)**. For typical head movements, patients’ peak velocities are within normal range for comparably sized head movements **(A)** but are significantly slower when larger head movements are attempted **(B)**. Circles are patient values. Dashed line is linear regression line for patients; solid line is linear regression line for control subjects with 95% confidence intervals (dotted lines).

Patients’ head movement amplitudes were significantly smaller than head movement amplitudes for controls when making typical head movements (*p* < 0.0001). When asked to make larger head movements, amplitudes increased significantly (*p* < 0.0001) (Table 1). However, compared to healthy subjects, patients still made smaller head movements even when intentionally increasing head movement amplitudes (*p* < 0.0001). For head movements of similar size, head durations were significantly longer for patients compared to controls for both typical (*F* = 11.69, *p* = 0.0009) and intentionally larger head movements (*F* = 15.62, *p* = 0.0002). Peak head velocities were not significantly different between healthy subjects and patients for the range of small head amplitudes made during typical head movements (*p* = 0.8579) (Figure 3A) but were significantly slower when larger amplitude head velocities were performed (*p* < 0.0001) (Figure 3B).

The contribution of head movements to total gaze amplitudes was significantly less for patients compared to controls for typical head movements (*p* < 0.0001) (Figure 1B). The mean ratio of head contribution to gaze amplitude for typical head movements was only 0.06 (SE 0.01), indicating that head movements contributed almost nothing to the total gaze displacements. Although the eye-in-orbit positions were not specifically measured prior to gaze shifts, patients’ eye–head kinematics were consistent with eye positions deviated away from the direction of the intended eye–head movements (16). When the eyes are deviated away from the direction of the intended gaze shift, the amplitude of eye-in-orbit movement in the direction of the intended gaze shift is extended. Therefore, the gaze shift can be made with a smaller head contribution (16), similar to the eye–head kinematics demonstrated for our patients making typical head-free saccades (Figure 1B). When consciously increasing head amplitudes, patients began to utilize head movements earlier in the course of the gaze saccades with increasing head and decreasing eye-in-orbit contributions, similar to normal subjects and consistent with greater alignment of orbital eye position with the direction of the impending gaze saccade (Figure 1C). With increasing head amplitudes, ratio of head contribution to total gaze amplitude increased significantly to 0.19 (SE 0.01) (*p* < 0.0001), similar to normal subjects making typical head movements, but much less than normal subjects consciously increasing head movement amplitudes (*p* = 0.0004) (Table 1).

Gaze saccade gains were significantly less for patients than for healthy subjects for typical head movements (*p* < 0.0001) (Table 1). Gaze accuracy improved significantly when patients made intentionally larger head movements (*p* < 0.0001) but was still less accurate than healthy subjects (*p* = 0.0422). Even including second saccades, patients had significantly smaller total gaze saccade gains for both typical (*p* < 0.0001) and larger head movements (*p* < 0.0001). For typical head movements, gaze saccade asymptotic peak velocities were slower; however, these differences did not reach statistical significance (*p* = 0.1518) (Table 2) and gaze saccade durations were not prolonged compared to controls (*F* = 0.1452, *p* = 0.7039). When making larger head movements, asymptotic gaze peak velocities were significantly slower (*p* = 0.0435) (Table 2) and gaze saccade durations were significantly prolonged for patients compared to healthy subjects (*F* = 24.93, *p* < 0.0001). Gaze saccade latencies were prolonged in patients compared to controls (*p* < 0.0001), did not change when making larger head movements (*p* = 0.4907), and remained significantly prolonged compared to healthy subjects (*p* < 0.0001). There was also significant prolongation of head onset relative to gaze onset for patients compared to controls for typical head movements (*p* = 0.0355) (Table 1). Head onset was significantly faster relative to saccade onset when patients made larger head movements (*p* < 0.0001), and there was no difference between head onset relative to saccade onset for patients compared to controls for larger head movements (*p* = 0. 5989). That is, with increasing head amplitudes, head movements began faster relative to gaze onset, but there was no relationship between gaze amplitude and head onset relative to gaze onset, as occurred in healthy subjects for either typical (Spearman *r* = −0.0491, *p* = 0.7407) or increased (Spearman *r* = −0.1479, *p* = 0.2511) head movement amplitudes.

Vestibulo-ocular reflex gains at the end of the first gaze saccade were significantly less for patients compared to controls for smaller, typical head movements (*p* < 0.0001) (Table 1; Figure 2B). Although VOR gains increased significantly when patients used larger head movements (*p* < 0.0001), patients continued to show greater VOR suppression compared to controls (*p* < 0.0001) (Figure 2C). Unlike normal subjects, there was no correlation between VOR gains and gaze saccade amplitudes or gaze saccade gains for either typical or larger head movements, even when taking second saccades into account. Likewise, VOR suppression did not negatively correlate with gaze saccade duration or latency.

## Discussion

We examined individuals who have pain and discomfort in their necks and have reduced head movement amplitudes, presumably to prevent further pain. This is a circumstance that occurs in many individuals; soft tissue pain can result from postural abnormalities, trauma, or arthritis. None of the individuals tested had vestibular loss, cerebellar dysfunction, cervical dystonia, or structural damage to their necks; their pain was the result of diminished soft tissue compliance and myalgia. However, as a group and consistent between individuals, these patients showed enhanced VOR suppression, inaccurate gaze saccades, and altered eye–head kinematics, including delayed head and gaze onsets and prolonged durations. The alteration in the kinematics of eye–head saccades may be consistent with increased neck soft tissue viscosity and stiffness, mimicking increased head moment of inertia. Likewise, there may be impaired feedback from neck proprioceptors to more rostral brainstem or cerebellar structures involved in VOR suppression and maintenance of gaze accuracy.

When there is greater head moment of inertia, head movements show decreased peak velocity and have longer durations resulting in longer, slower gaze saccades (3). Also, with greater head inertia, the head contribution to the gaze saccade decreases and the eye-in-orbit contribution increases. This is consistent with the proposition that gaze and head trajectories, but not eye movements, are controlled through two feedback loops (2). These are adaptive strategies designed to optimize the dynamics of combined eye–head movements and minimize noise, which can compromise accuracy. Similar to situations of increased head inertia, patients’ head movements also had longer durations for both typical and intentionally larger head amplitudes. With typical head movements, patients’ peak head velocities were similar to controls but only within the very small range of head movement amplitudes, and only a very small portion (6%) of gaze amplitude was due to head movement. When asked to make larger head movements, both head and gaze velocities were significantly slower. Thus, patients with decreased neck soft tissue compliance have altered eye–head kinematics similar to the strategies used by individuals with increased head inertia to reduce gaze error (3). However, in our patients, these kinematics do not yield accurate gaze saccades.

A number of studies have looked at how the VOR is suppressed when making large eye–head (gaze) saccades. This has been examined in normal subjects or non-human primates by unexpectedly braking the head during head-free (1, 7) or head-restrained movements (4). None of these manipulations have resulted in reduced gaze accuracy. Instead, they have shown that gaze saccades remain precise regardless of changes in head movement. Daye et al. (4) proposed an internal gaze command which plans a head movement, but does not necessarily evoke that head movement, and which can also alter the VOR. The combined action of both vestibular input and a gaze feedback loop is designed to reduce gaze error and ensure that the gaze lands on target. If the VOR is left on, signals to suppress gaze burst neuron activity slow eye movements and allow the head to contribute to gaze displacement. Therefore, it has been proposed that the degree of VOR suppression varies with gaze saccade duration (2). This was apparent in healthy subjects. Instead, the VOR in patients was suppressed at the end of the first saccade and remained suppressed until the end of the second saccade (Figure 2B). VOR suppression did not correlate with gaze saccade duration. Even with an additional saccade, gaze accuracy was not as precise in patients as for control subjects and gaze saccade gains did not correlate with VOR suppression, although generally speaking, greater gaze accuracy occurred when there was less VOR suppression. This suggests that there is a mismatch between the internal representation of head movement and the actual head movement information received from the semicircular canals (2). If the internal representation of head movement corresponds to a larger movement than the actual movement, the gaze controller will stop its action because the sum of internal head and eye displacements will be equal to the desired gaze displacement. This will lead to undershot, inaccurate gaze saccades, like the ones observed in our patient population.

Another essential component in the coordination of eye–head shifts is the position of the eyes in the orbits at the start of the gaze movement (3, 16). The position of the eye in the orbit will influence both the size of the head movement and its onset relative to the size of the gaze saccade. Patients delayed the onset of both gaze and head movements and reduced the amplitudes of the initial gaze saccades (Figure 1B), likely related in part to deviation of the eye-in-orbit away from the intended direction of gaze shift (3, 16). If the eye-in-orbit is deviated away from the direction of the intended gaze shift, the eye contribution to the amplitude of the gaze movement is much greater than the head contribution, which is delayed. In our patients, the head component to the gaze saccade was planned by the internal gaze command but was not initiated until the eye-in-orbit neared its mechanical limit and had not achieved target acquisition.

With intentionally larger head movements, head onset latency was reduced and patients began using the more typical two-phase approach to acquire a peripheral target; that is, a larger eye–head saccade during which the VOR is completely suppressed followed by counter-rotation of the eye-in-orbit using an active VOR to maintain gaze fixation (Figure 2C). This would also cause the eye-in-orbit to be more aligned with the direction of the subsequent gaze shift, thereby initiating the head contribution sooner and increasing its contribution. Although improved, head movement amplitudes were still reduced, the VOR remained suppressed and gaze saccade accuracy continued to be impaired.

While the vestibular system is designed to stabilize the gaze, the neck proprioceptive system is designed to stabilize head-on-trunk movement. Patients with axial rigidity secondary to dystonia or parkinsonism show increased dampening of pitch responses to linear acceleration, which has been attributed to increased viscosity of the neck tissues (18). With voluntary neck stiffening, Peng et al. (19) have modeled an increased tendency to stabilize the head on the trunk and oppose head stabilization in space for yaw movements, similar to pitch responses with increased viscosity. Like our patients, patients with torticollis delay the onset of both gaze and head movements but continue to show normal saccade dynamics, including accuracy (20). Patients with neck muscle pain may also have increased cervical muscle stiffness and viscosity, which would tend to increase head-on-trunk stabilization and contribute to reduced head movement amplitudes during non-predictable gaze shifts. EMG studies of neck muscles of normal subjects, who were asked to stabilize their weighted heads against an applied load, produced neck muscle co-contraction which appeared to be designed to stabilize both neck muscles and head (21). In patients with chronic neck pain, similar EMG studies of neck muscle activation during weighted arm movement tasks have shown reduced cervical muscle velocities and accelerations and prolonged coactivation of cervical muscles during the task (22). The alteration in eye–head kinematics which resembles increased head moment of inertia may be caused by these changes in cervical muscle and soft tissue dynamics and abnormal, prolonged coactivation of cervical muscles and are unlikely to be the result of attempts to minimize gaze error. Slower, smaller head movements maintain head-on-trunk stability at the expense of gaze accuracy. Recently, Brandt and Huppert (8) have proposed this same mechanism as a cause of “cervical vertigo,” whereby neck muscle stiffness causes head movements to be smaller than intended.

Roy and Cullen (5) found a VOR cancelation signal to the vestibular nuclei occurred only when activation of neck proprioceptors matched the neck motor command. In patients with increased neck stiffness, the VOR is suppressed; therefore, the command to move the neck must theoretically match the neural output from the neck proprioceptors. That is, the head movement initiated by the motor command to the neck muscles is smaller than programed because of increased neck muscle viscosity and stiffness, thereby resulting in a smaller vestibular signal. However, proprioceptive signals from neck muscles are larger because of ongoing coactivation of cervical muscles. These signals match the model’s estimate of the head movement, thereby suppressing the VOR at the level of the vestibular nuclei.

Finally, the patients described by Brandt and Huppert (8) had dizziness (cervical vertigo) due to acute neck pain and resolving when the pain subsided. Our patients had longstanding chronic neck pain and restricted head movements. It is possible that their enhanced VOR suppression is a function of the chronicity of their disorder and the result of anti-Hebbian plasticity in the cerebellum which, over time, induces synaptic changes to cause further reduction in the vestibular response to head movement (23).

The practical consequences to the patient may be dizziness in the short term (8) and imbalance and falls in the long term. Simplistically, improving eye–head kinematics and gaze accuracy by voluntarily increasing head movement amplitudes and head contributions to gaze saccades may lessen symptoms, improve stability, and reduce falls. In order to prevent secondary plastic changes, treatment should be aimed at improving neck mobility as soon as possible. Prospective studies of gaze accuracy should be undertaken to assess therapies designed to improve neck mobility, particularly in relation to falls and to subjective symptoms of dizziness and imbalance.

## Conclusion

Patients with chronic restriction of neck movements due to pain produce inaccurate combined eye–head saccades. An internal gaze command plans a combined eye–head movement and modification of vestibular input in order to create an accurate gaze saccade. The command is issued for a head movement with large enough amplitude to create an accurate gaze saccade; however, because of increased neck stiffness and viscosity, the head movement is smaller, slower, longer, and more delayed than it should be. Likewise, VOR suppression is disproportionate to the size of the actual gaze saccade because sensory feedback signals from neck proprioceptors are non-veridical, likely due to prolonged coactivation of cervical muscles. The combination of a small head contribution to the gaze shift and prolonged, amplified VOR suppression may impair cerebellar control of gaze shift accuracy in the absence of the VOR. The outcome of these alterations in eye–head kinematics is head-on-trunk stability at the expense of gaze accuracy during head-free gaze shifts. Future studies could address gaze kinematic changes that occur with increased head inertia in individuals with restricted cervical motion.

## Ethics Statement

This study was carried out in accordance with the recommendations of the University of Manitoba Policy 1406 (The Ethics of Research Involving Humans) and the Tri-Council Working Group on Ethical Conduct for Research Involving Human Subjects as per the University of Manitoba Ethics Committee with written informed consent from all the subjects. Potential side effects of magnetic search coil oculography including corneal abrasion, blurred vision, and ocular irritation were discussed with patients and healthy subjects prior to ocular motor testing by one author (Janine L. Johnston). Each subject was carefully examined prior to testing to assure that there were no physical impediments to undergoing the test protocol. All the subjects gave written informed consent in accordance with the Declaration of Helsinki. The protocol was approved by the University of Manitoba Ethics Committee.

## Author Contributions

JJ developed the experimental paradigms, undertook the ocular motor recordings, and processed the data. GT recruited and examined the patients to assure that they met inclusion criteria for the study. JJ and PD interpreted the data. JJ, PD, and GT drafted the manuscript, critically reviewed it for intellectual content, and have edited and approved the final version to be published.

## Conflict of Interest Statement

The authors declare that the research was conducted in the absence of any commercial or financial relationships that could be construed as a potential conflict of interest.
